# Advancing PAM-less genome editing in soybean using CRISPR-SpRY

**DOI:** 10.1093/hr/uhae160

**Published:** 2024-06-07

**Authors:** Xiao Chen, Zhaohui Zhong, Xu Tang, Suxin Yang, Yaohua Zhang, Shoudong Wang, Yiqian Liu, Ye Zhang, Xuelian Zheng, Yong Zhang, Xianzhong Feng

**Affiliations:** Key Laboratory of Soybean Molecular Design Breeding, National Key Laboratory of Black Soils Conservation and Utilization, Northeast Institute of Geography and Agroecology, Chinese Academy of Sciences, Changchun 130102, China; Department of Biotechnology, School of Life Sciences and Technology, Center for Informational Biology, University of Electronic Science and Technology of China, Chengdu 610054, China; Department of Biotechnology, School of Life Sciences and Technology, Center for Informational Biology, University of Electronic Science and Technology of China, Chengdu 610054, China; Integrative Science Center of Germplasm Creation in Western China (Chongqing) Science City, Chongqing Key Laboratory of Plant Resource Conservation and Germplasm Innovation, School of Life Sciences, Southwest University, Chongqing 400715, China; Key Laboratory of Soybean Molecular Design Breeding, National Key Laboratory of Black Soils Conservation and Utilization, Northeast Institute of Geography and Agroecology, Chinese Academy of Sciences, Changchun 130102, China; College of Advanced Agricultural Sciences, University of Chinese Academy of Sciences, Beijing 100049, China; Key Laboratory of Soybean Molecular Design Breeding, National Key Laboratory of Black Soils Conservation and Utilization, Northeast Institute of Geography and Agroecology, Chinese Academy of Sciences, Changchun 130102, China; Key Laboratory of Soybean Molecular Design Breeding, National Key Laboratory of Black Soils Conservation and Utilization, Northeast Institute of Geography and Agroecology, Chinese Academy of Sciences, Changchun 130102, China; College of Life Sciences, Jilin Agricultural University, Changchun 130118, China; Key Laboratory of Soybean Molecular Design Breeding, National Key Laboratory of Black Soils Conservation and Utilization, Northeast Institute of Geography and Agroecology, Chinese Academy of Sciences, Changchun 130102, China; College of Advanced Agricultural Sciences, University of Chinese Academy of Sciences, Beijing 100049, China; Department of Biotechnology, School of Life Sciences and Technology, Center for Informational Biology, University of Electronic Science and Technology of China, Chengdu 610054, China; Integrative Science Center of Germplasm Creation in Western China (Chongqing) Science City, Chongqing Key Laboratory of Plant Resource Conservation and Germplasm Innovation, School of Life Sciences, Southwest University, Chongqing 400715, China; Department of Biotechnology, School of Life Sciences and Technology, Center for Informational Biology, University of Electronic Science and Technology of China, Chengdu 610054, China; Integrative Science Center of Germplasm Creation in Western China (Chongqing) Science City, Chongqing Key Laboratory of Plant Resource Conservation and Germplasm Innovation, School of Life Sciences, Southwest University, Chongqing 400715, China; Key Laboratory of Soybean Molecular Design Breeding, National Key Laboratory of Black Soils Conservation and Utilization, Northeast Institute of Geography and Agroecology, Chinese Academy of Sciences, Changchun 130102, China; College of Advanced Agricultural Sciences, University of Chinese Academy of Sciences, Beijing 100049, China

## Abstract

Although CRISPR-Cas9 technology has been rapidly applied in soybean genetic improvement, it is difficult to achieve the targeted editing of the specific loci in the soybean complex genome due to the limitations of the classical protospacer adjacent motif (PAM). Here, we developed a PAM-less genome editing system mediated by SpRY in soybean. By performing targeted editing of representative agronomic trait targets in soybean and evaluating the results, we demonstrate that the SpRY protein can achieve efficient targeted mutagenesis at relaxed PAM sites in soybean. Furthermore, the SpRY-based cytosine base editor SpRY-hA3A and the adenine base editor SpRY-ABE8e both can accurately induce C-to-T and A-to-G conversion in soybean, respectively. Thus, our data illustrate that the SpRY toolbox can edit the soybean genomic sequence in a PAM-free manner, breaking restrictive PAM barriers in the soybean genome editing technology system. More importantly, our research enriches soybean genome editing tools, which has important practical application value for precise editing and molecular design in soybean breeding.

## Introduction

In plant genome editing, CRISPR-Cas9 technology has emerged as a pivotal tool, holding the potential to revolutionize crop genetic improvement with its precision gene editing and regulatory capabilities [1]. However, the conventional CRISPR-Cas9 system necessitates specific protospacer adjacent motif (PAM) sequences as recognition sites, especially NGG PAMs for *Streptococcus pyogenes* Cas9 (SpCas9). This requirement not only restricts the selection of target sites but also affects gene editing efficiency and system adaptability, posing a major challenge for current genome editing applications in crop improvement.

**Figure 1:**
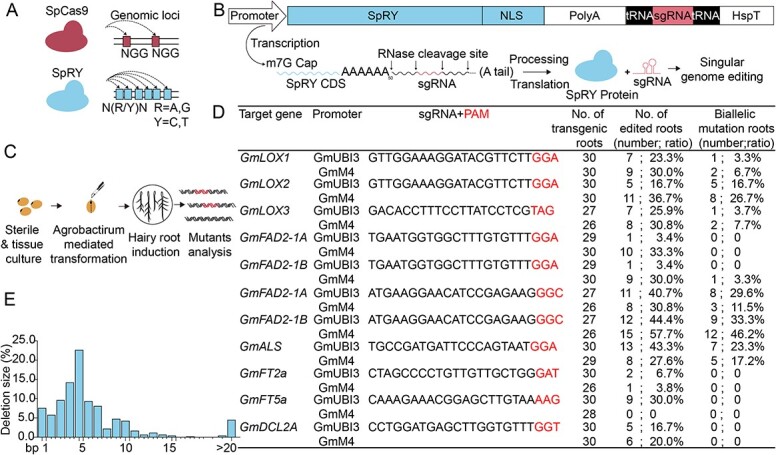
Analysis of SpRY nuclease activity on different PAMs in soybean hairy roots. **A** Illustration of SpCas9 and SpRY proteins. SpCas9 mainly recognizes NGG PAM sites, whereas SpRY recognizes N(R/Y)N PAM sites. **B** Schematic of the soybean SpRY system based on the STU-Cas9 2.0 system. The single transcript unit is driven by the Pol II promoter, and sgRNAs are released by tRNA processing, enabling singular genome editing. **C** Overview of the stepwise process of genome editing in soybean hairy roots using SpRY. **D** Summary of genome editing efficiency by SpRY at 11 endogenous target sites in soybean hairy roots. **E** Deletion size profile of SpRY in soybean hairy roots.

Recently, an engineered variant SpRY has largely overcome these PAM-related limitations [2]. This breakthrough has significantly enhanced genome editing capabilities, enabling highly efficient and precise gene editing as well as base editing in human cells, almost without any PAM restrictions. In particular, the NRN PAM sites are more efficient than NYN PAM sites (Fig. 1A) [2]. Consequently, SpRY holds the potential to revolutionize plant genome editing. Now, SpRY has successfully demonstrated its effectiveness in genome editing within plant species such as rice [3,4] and Dahurian larch [4]. The emergence of SpRY not only extends the editing capabilities of the CRISPR-Cas system within the genome but also paves the way for novel avenues in plant functional genomics research and crop breeding.

Soybean (*Glycine max* (L.) Merr.), a vital economic diploid crop derived from a paleotetraploid ancestor, provides abundant protein and oil for both humans and animals [5]. At present, the main reason for restricting soybean production is the lack of high-yield and high-quality varieties. Traditional breeding methods have reached their limits in improving soybean yield and quality and cannot meet the needs of the future growing population. Therefore, accelerating the application of soybean biotechnology breeding is a crucial strategy to solve the bottleneck of soybean production. Despite the opportunities offered by CRISPR-Cas technology to enhance crucial agronomic traits and expedite breeding cycles in soybean [6–16], prevailing genome editing systems still grapple with technical challenges like low target efficiency, PAM constraints, and complexities of single-base substitutions, limiting their scope [17–18]. What is more noteworthy is that there are still many agronomic trait-related loci that are currently unavailable for precise genome editing.

Some soybean genes that control important agronomic traits have been cloned and can be used as targets for gene editing to generate more useful new germplasm resources. Three lipoxygenases (LOXs), GmLOX1, GmLOX2, and GmLOX3, have been proved as effective targets to remove the unpleasant beany taste in soybean seeds, which involves catalyzing polyunsaturated fatty acids to hydroperoxides [19–21]. *GmFAD2-1A* and *GmFAD2-1B* are commercial targets to significantly increase the content of oleic acid for superior oil quality, which catalyzes the conversion of oleic acid to linoleic acid [22–23]. *GmFT2a* and *GmFT5a*, two important *FLOWERLOCUST* (*FT*) genes, have been widely identified and reported as flowering activators regulating the flowering time to improve the regional adaptability of soybean [24, 25]. In addition, the acetolactate synthase (*ALS*) [26] and 5-enolpyruvylshikimate-3-phosphate synthase (*EPSPS*) [27] genes are the targets of herbicides widely used for weed control in fields. The generation of new alleles related to herbicide resistance has important applications in agriculture. Previous studies have already edited these genes using CRISPR-Cas9 to create edited plants with altered or improved phenotypes [10, 25, 28]. Developing soybean PAM-less genome editing tools will further accelerate the soybean breeding process through editing the above important genes.

In this study we applied SpRY to soybean genome editing to achieve efficient editing at non-PAM sites for the first time, and developed base editing tools, thereby expanding the applicability of the CRISPR system in soybean genome editing. The important agronomic trait genes, such as *GmLOX*s, *GmFAD2-1A/1B*, *GmFT2a*, and *GmFT5a*, were selected as target genes to detect SpRY activity in soybean. Our research will provide a powerful tool for accurate targeted editing and base editing of arbitrary DNA sequences in the whole soybean genome. Meanwhile, it will also have important theoretical and practical application value for soybean genetic improvement, and lays a theoretical and technical foundation for precise editing and molecular design in soybean breeding.

## Results

### Establishment of PAM-less genome editing system in soybean

Drawing from the effectiveness of the previously reported single transcript unit (STU)-Cas9 2.0 system in plant genome editing, which could use a single Pol II promoter for both Cas9 and sgRNA expression [29], we adopted the approach to construct the soybean SpRY system based on the STU-Cas9 2.0 system (Fig. 1B). Two highly expressed constitutive promoters, GmUBI3 [30] and GmM4 [31, 32], were employed to drive the expression of the SpRY nuclease and guide RNA. SpRY’s performance in soybean was assessed by targeting non-NGG PAM loci associated with agronomic traits in soybean hairy roots (Fig. 1C). The editing efficiency was evaluated by Sanger sequencing of PCR products. The data showed that the GmUBI3- and GmM4-driven SpRY systems both demonstrated effective genome editing at a variety of soybean loci, achieving efficiencies of up to 57.7% (Fig. 1D; Supplementary Data Figs S1–S3). Furthermore, SpRY was found to induce frequent 3- to 7-bp deletions at these target loci in soybean (Fig. 1E), which are consistent with those in rice, and this feature holds promise for editing regulatory elements and non-coding RNA regions [4].

### Evaluation of off-targeting frequency of SpRY in soybean

**Figure 2:**
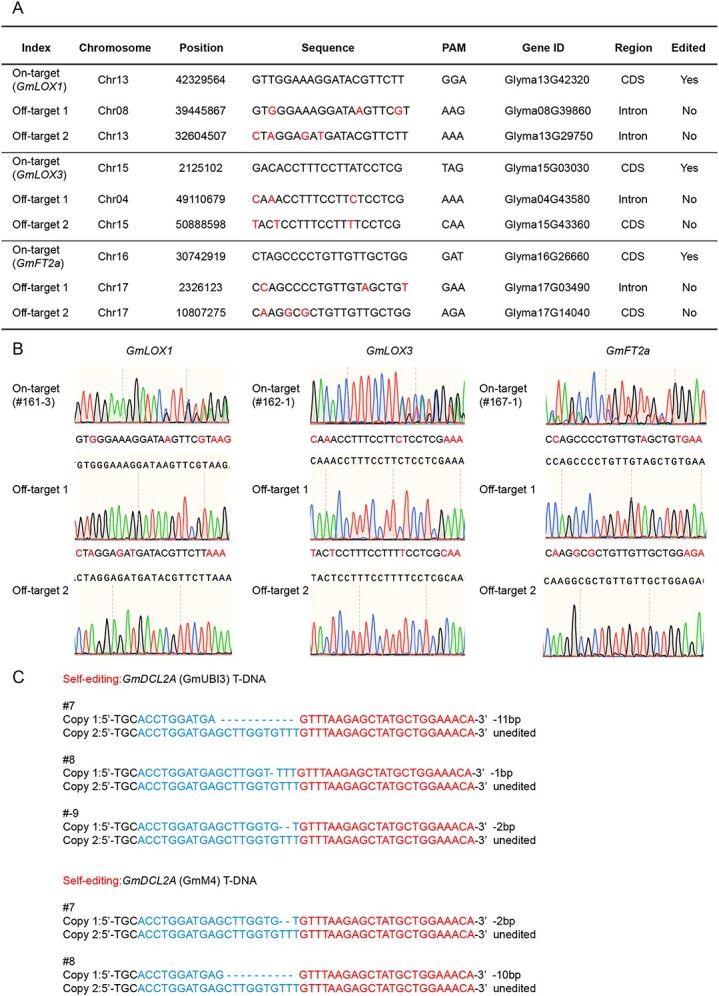
Off-target assessment of SpRY nuclease in soybean hairy roots. **A** Summary of off-target sites predicted by CRISPR-GE software. **B** Sanger sequencing results of on-target site and off-target sites. All off-target sites showed that no editing event happened. **C** T-DNA self-cleavage induced by SpRY in soybean hairy roots. The protospacer is highlighted in blue. The gRNA scaffold is highlighted in red.

Due to the PAM-less properties of SpRY, off-targeting could be caused by sequence similarity to the targets and the self-editing of T-DNA [4]. Based on likely off-target sites predicted by Cas-OFFinder [33], we conducted on-target and off-target test for the newly developed soybean SpRY systems targeting GGA, TAG, and GAT PAMs to determine the probability of off-targeting in soybeans (Fig. 2A).

Sanger sequencing results revealed that while all the three designed on-target sites were edited, the six predicted off-target sites still maintained the wild-type sequences, indicating that there were no off-target mutations at these examined sites (Fig. 2B) Additionally, we found that the soybean SpRY editing system also exhibited T-DNA self-editing at specific sites (Fig. 2C). Notably, these sites displayed reduced genome editing efficiency (Supplementary Data Fig. S4), suggesting a potential correlation that provides insights into strategies for enhancing the soybean SpRY system in the future.

### Evaluation of multiplex gene editing efficiency of SpRY in soybean

**Figure 3:**
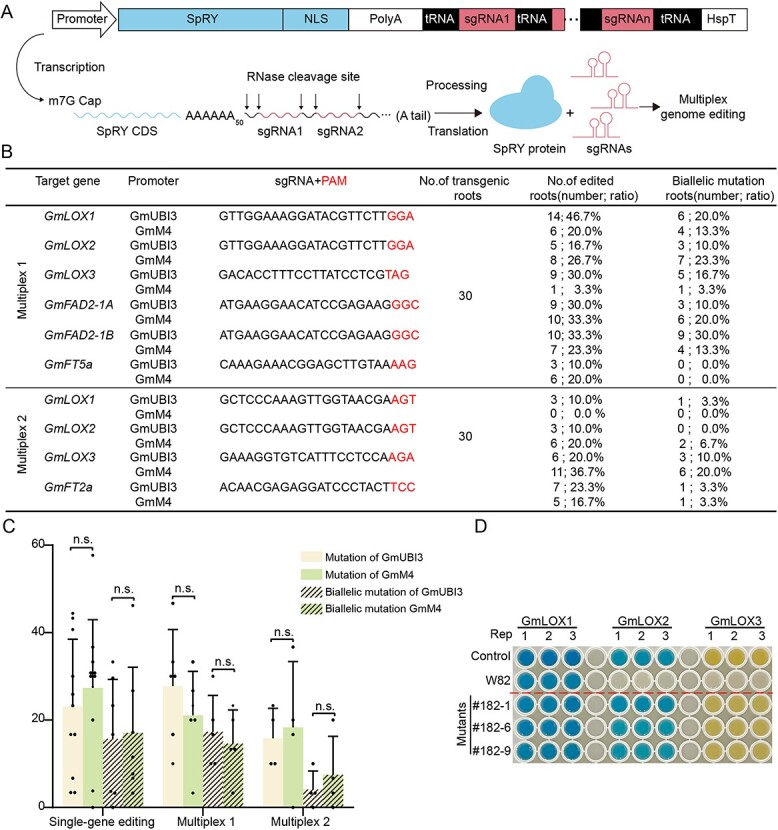
Analysis of SpRY-mediated multiplex gene editing efficiency at different PAMs in soybean. **A** Schematic of the soybean SpRY system based on the STU-Cas9 2.0 system. The single transcript unit was driven by the Pol II promoter, and sgRNAs were released by tRNA processing, enabling multiplex genome editing. **B** Summary of multiplex genome editing efficiency by SpRY in soybean hairy roots. **C** Statistical analysis of mutation efficiency and biallelic mutation efficiency of the GmUBI3 and GmM4 systems among the tested target sites in soybean hairy roots. The pale yellow column represents the mutation efficiency of GmUBI3; the pale green column represents the mutation efficiency of GmM4; the pale yellow striped column represents the biallelic mutation efficiency of GmUBI3; and the pale green striped column represents the biallelic mutation efficiency of GmM4. The data were analyzed using the unpaired *t*-test with two-tailed *P* value. Each dot represents a biological replicate, *P* > 0.05, n.s. (not significant). **D** Assessment of lipoxygenase activity of multiplex edited hairy roots of the GmLOX1/2/3 by colorimetric assay.

To further validate SpRY’s multiplex gene editing in soybean, we constructed two SpRY multiplex vectors targeting six and four sites, respectively (Fig. 3A). Gene editing activity was also evaluated in soybean hairy roots. Our results showed that SpRY is capable of achieving multiplex gene editing in soybean (Fig. 3B; Supplementary Data Figs S5–S7). On further statistical analysis, the average mutation efficiency and biallelic mutation efficiency of GmUBI3 for single-gene editing were 23.14 and 15.70%, for six-gene editing they were 27.78 and 17.34%, and for four-gene editing they were 15.83 and 4.15%, respectively. For GmM4s, the corresponding average mutation efficiency and biallelic mutation efficiency were 27.34 and 17.04% for single-gene editing, 21.10 and 14.64% for six-gene editing, 18.35 and 7.50% for four-gene editing, respectively. Statistical analysis of the data using unpaired *t*-tests with two-tailed *P*-values indicated that the two promoters have considerable activities in soybeans (Fig. 3C). Notably, both systems successfully generated hairy roots with biallelic mutations (Figs 1D and 3B). Meanwhile, we successfully obtained hairy roots with knockout of the *GmLOX1/2/3* genes (Supplementary Data Fig. S5). We examined the lipoxygenase activity of *GmLOX1/2/3* using a colorimetric assay in the knockout mutation materials. It is possible that GmLOX1 is highly expressed in seeds, so we did not detect its activity in soybean hairy roots (Fig. 3D). However, we confirmed that the activity of GmLOX2 (indicated by the remaining blue solution) and GmLOX3 (the remaining yellow solution) was absent in *GmLOX1/2/3* knockout soybean hairy roots (Fig. 3D). Overall, both systems demonstrated effective SpRY genome editing capabilities in soybean. More importantly, we developed the PAM-less SpRY multiplex gene editing system based on the STU-Cas9 2.0 system, which is helpful to study the regulatory pathways for important agronomic traits genes.

### Realization of PAM-less base editing in soybean

**Figure 4:**
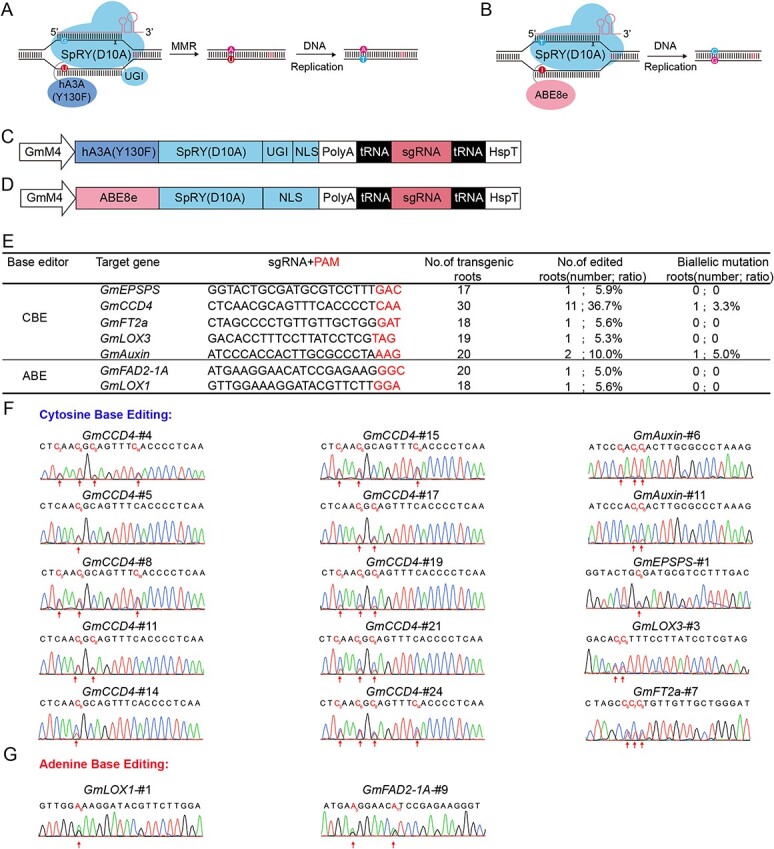
Analysis of the SpRY-mediated editing activity of the base editors on different PAM sites in soybean hairy roots. **A** Schematic of the SpRY-mediated mechanisms of cytosine base editing. **B** Schematic of the SpRY-mediated mechanisms of adenine base editing. **C** Schematic of the SpRY cytosine base editor systems in soybean. **D** Schematic of the SpRY adenine base editor systems in soybean. ABE, adenine base editor. **E** Summary of base editing efficiency by SpRY at endogenous sites in soybean hairy roots. CBE, cytosine base editor. **F** Sanger sequencing results of cytosine base editing by SpRY in soybean hairy roots. Red arrows indicate conversion, and the positions of mutated nucleotides are labeled above with corresponding numbers. **G** Sanger sequencing results of adenine base editing by SpRY in soybean hairy roots. Red arrows indicate conversion, and the positions of mutated nucleotides are labeled above with corresponding numbers.

The STU system has previously demonstrated the capability to achieve base editing through various deaminase domains [29]. Hence, we sought to confirm the feasibility of implementing SpRY-based base editing in soybean. By combining hA3A_Y130F [34] and ABE8e [35] deaminase domains, we engineered cytosine and adenine base editors, respectively, both under the control of the GmM4 promoter for expression (Fig. 4A–D).

We assessed the editing activity of cytosine base editors at five sites and adenine base editors at two sites using soybean hairy roots (Fig. 4E). For the cytosine base editor, C-to-T conversion was detected at the target sequence of *GmEPSPS*-GAC, *GmCCD4*-CAA, *GmFT2a*-GAT, *GmLOX3*-TAG, and *GmAuxin*-AAG based on the sequencing results (Fig. 4F). The cytosine base editing (CBE) activity ranged from 5.3 to 36.7%. Among the activities, the editing efficiency of C to T in the *GmCCD4*-CAA target site is relatively high. Furthermore, indels were also detected at the *GmCCD4*-CAA site. For the adenine base editor, A-to-G conversion was detected at the target sequence of *GmFAD2-1A*-GGC and *GmLOX1*-GGA. The adenine base editing (ABE) activity ranged from 5.0 to 5.6%. Although the editing efficiency of SpRY-ABE8e in soybean is very low, the data indicated that SpRY-ABE8e is capable of efficiently inducing A-to-G conversions toward non-canonical PAM sites (Fig. 4G). The above results demonstrated that SpRY can indeed achieve cytosine and adenine base editing. Moreover, we successfully obtained biallelic C-to-T edits (Fig. 4E). These findings further expand the potential applications of SpRY in soybean.

### Application of SpRY protein in soybean high oleic acid germplasm generation

**Figure 5:**
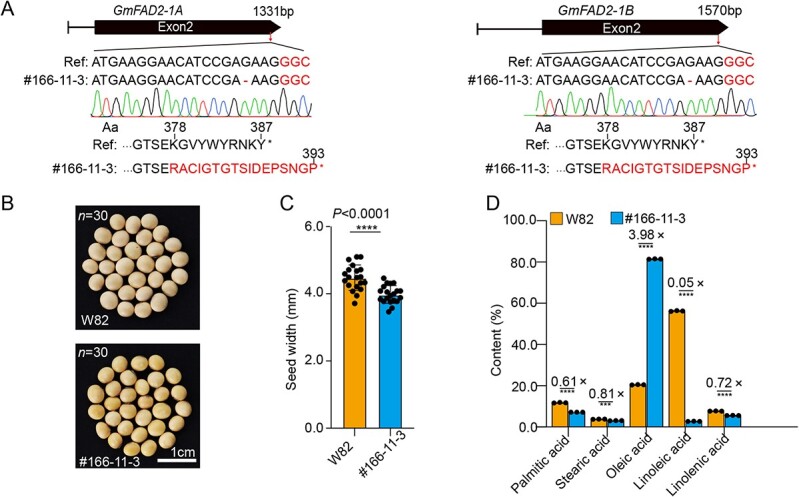
SpRY induced *GmFAD2-1A/1B* mutation in soybeans. **A** Schematics of *GmFAD2-1A* and *GmFAD2-1B* and genotypes of *GmFAD2-1A/1B* double mutant *T*_2_ line #166-11-3. **B** Seeds of wild-type ‘Williams 82’ and *GmFAD2-1A/1B* double mutant *T*_2_ line #166-11-3. **C** Statistical analysis of seed width between wild-type ‘Williams 82’ and *GmFAD2-1A/1B* double mutant *T_2_* line #166-11-3. **D** Fatty acid contents of seeds in wild-type ‘Williams 82’ and *GmFAD2-1A/1B* double mutant *T*_2_ line #166-11-3. Data were analyzed using the unpaired *t*-test with two-tailed *P* value. Each dot represents a biological replicate. ^*^^*^^*^P < 0.001; ^*^^*^^*^^*^P < 0.0001.

To determine whether SpRY was able to produce stably edited plants in soybean, we transformed the T-DNA vector containing *GmFAD2-1A/1B*-GGC sgRNA with high targeting efficiency in hairy roots into the cultivar ‘Williams 82’ (W82) by *Agrobacterium*-mediated transformation. We obtained 30 *T*_0_ independent transformation lines, of which 17 were PCR-validated as transgene-positive plants. A total of 10 edited plants were obtained by detecting the *GmFAD2-1A/1B*-GGC target site. Among these edited plants, we obtained eight *Gmfad2-1a/1b* double knockout plants, one *Gmfad2-1a* knockout plant, and one *Gmfad2-1b* knockout plant. The editing efficiency of SpRY at the *GmFAD2-1A/1B*-GGC target sites was 58.8%. Furthermore, we conducted an analysis of agronomic traits and fatty acid content in the seeds of *T*_2_  *Gmfad2-1a/1b* homozygous edited plants. We obtained a homozygous double mutant line, #166-11-3, with a 1-bp deletion at the *GmFAD2-1A* and *GmFAD2-1B* target sites, respectively (Fig. 5A; Supplementary Data Fig. S8A and B). It is worth noting that this mutation causes the GmFAD2-1A and GmFAD2-1B proteins in homozygous double mutant to have six more amino acids in the C-terminal (Fig. 5A). We observed that the seeds of the homozygous double mutant were significantly smaller than those of the wild type (Fig. 5B). Therefore, we compared the seed length, width and thickness of the mutant and the wild type. We found that there was a significant difference between them in terms of seed width (Fig. 5C; Supplementary Data Fig. S8C). Then, the fatty acid components in the double mutant seeds were detected by gas chromatography. The results showed that the oleic acid content of the double mutant #166-11-3 was significantly increased from 20.6 to 81.4%, while the linoleic acid content decreased significantly from 56.3 to 2.84%. The other three fatty acids, palmitic acid, stearic acid, and linolenic acid, also decreased (Fig. 5D; Supplementary Data Fig. S8D), which is consistent with the previous reports [10]. This work not only further validates the ability of SpRY for PAM-less genome editing in soybean, but also provides new germplasm resources for high oleic acid soybeans.

## Discussion

The emerging biotechnology represented by CRISPR-Cas technology can efficiently and accurately induce mutations at specific target sites, making it a powerful tool for crop genetic improvement, and bringing new revolutions for modern crop genetic breeding [36, 37]. However, compared with other crops, the existing genome editing system in soybean is still faced with technical bottlenecks such as PAM sequence restriction, low targeting efficiency, and inability to accurately achieve single-base replacement, leading to the limitation of its use. What is more noteworthy is that a ABE base editor has not been reported in soybean. Therefore, accelerating innovation in soybean genome editing technology system is one of the preconditions for making full use of genome editing technology to improve the quality of soybean and release the potential for increasing soybean yield.

In this study, we achieved PAM-less genome editing in soybean using SpRY nuclease based on the STU-Cas9 2.0 system. By detecting different PAM sites, it can be proved that SpRY nuclease can effectively edit soybean endogenous genes in a PAM-free manner, while Cas9 only shows high editing efficiency at typical NGG PAM sites compared with SpRY (Supplementary Data Fig. S9). In addition, our data showed that SpRY nuclease can achieve efficient targeted editing of a wide range of loci in the soybean genome with a preference for NRN PAM (NGN and NAN) sites, which is consistent with results in rice. Among them, at NGN PAM sites, SpRY roughly exhibited a high editing efficiency and displayed better editing at NGC PAM sites than at NGA, NGT, and NGG PAM sites. Moreover, SpRY nuclease is also able to target editing at the NYN locus in soybean, such as the TCC target site, while the editing efficiency is relatively not as high as at NRN sites. Although the editing efficiency of this system in soybean is not as high as that in rice, compared with other Cas variants introduced in soybean, such as xCas9, SpCas9-NG, XNG-Cas9, and Cas12a (Cpf1) [17, 18], the SpRY protein can achieve efficient editing of any site without PAM restriction, greatly expanding the editing scope of the soybean genome. What is more, there are no other Cas proteins with this property so far. Meanwhile, we also tried to use different promoters to improve the editing efficiency of SpRY based on previous studies [32, 38]. However, the results showed that there was no significant difference in editing efficiency between the GmM4 promoter reported to have high expression activity and the GmUBI3 promoter. This result indicated that we need to optimize the system in other ways to improve its editing efficiency in the future. In addition, we conducted the detection of self-editing of some test targets. It is worth noting that there seems to be a negative correlation between editing efficiency and self-editing efficiency in our study. Although self-editing occurs in soybeans, we did not find strong evidence for off-target effects of SpRY in soybeans. These results were consistent with those in rice [4].

Moreover, in terms of base editing, only three articles have reported the cytosine base editor in soybeans. However, there are no reports on the application of ABE base editors in soybean. The editing efficiency of soybean the cytosine base editor based on previous SpCas9 ranged from 6 to 18.2% [39]. Although using the SpCas9-NG variant [40] and optimized deaminase [41] can achieve >60% editing efficiency in hairy roots, soybean cytosine base editors are still limited by the PAM sites. Fortunately, we have effectively solved the bottleneck in this study. Testing of the SpRY-hA3A base editor developed in this study for five target sites in soybean hairy roots demonstrated that the PAM-less cytosine base editor could perform C-to-T base editing, and the editing efficiency could reach 36.7% at *GmCCD4*-CAA sites, while the editing efficiency at the other four target sites was relatively low (5.3–10.0%). This result is consistent with the observation of Gao’s group that the activity of hA3A is limited in soybeans [41]. We also made the SpRY version of ABE8e, which showed detectable A-to-G editing at relaxed PAM sites in soybean hairy roots. Although the efficiency of A-to-G base editing is generally much lower than C-to-T base editing, our study breaks the lack of ABE base editors in soybean, providing optional tools for the improvement of important agronomic traits in soybean.

Furthermore, we successfully induced mutations in *GmFAD2-1A* and *GmFAD2-1B* using the SpRY system. Although the *GmFAD2-1A/1B*-GGC target sites for *GmFAD2-1A* and *GmFAD2-1B* were located at the C terminus, we obtained homozygous double mutants with oleic acid content increased from 20.6 to 81.4% in this study. This result showed that C-terminal modification using the SpRY system affected the protein function of GmFAD2-1A and GmFAD2-1B. The reason for this result may be that the C terminus of the protein plays an important role in regulation of the protein activity or stability [42]. In summary, our work confirmed that SpRY protein can achieve PAM-free genome editing in soybean and provides new germplasm resources for soybean quality improvement breeding. The above results show that SpRY-mediated soybean genome editing system can effectively compensate for the sites that cannot be edited by traditional SpCas9, expanding the scope of soybean genome targeted editing, and providing effective tools for soybean whole-genome research. Although the editing efficiency at certain sites is relatively low, it is consistent with the characteristics of SpRY protein itself with preference for NR PAM sites. In summary, we successfully introduced a PAM-less SpRY genome editing system rooted in the STU-Cas9 strategy, amplifying its versatility through multiplex editing and proficient cytosine and adenine base editing. The SpRY toolbox surmounts PAM barriers in soybean, presenting a formidable tool for soybean gene discovery and molecular breeding.

## Materials and methods

### Plant materials and transformation

The soybean cultivar ‘Williams 82’ was used for transformation. The T-DNA vector was firstly transformed into *Agrobacterium rhizogenes* strain K599 for hairy root induction, and the hairy root induction procedure was as previously described [43]. Half of the hypocotyl was removed with a scalpel, and then the seed was cut along the umbilici to make the wound at the cotyledon node. The wounded cotyledons were placed in infection solution for 3 h. After infection, the explants were placed on solid co-cultivation medium (0.321 g/L B5 salt, 0.112 g/L B5, 0.59 g/L MES, 30 g/L sucrose, 8 g/L agar, 100 mg/L acetosyringone , pH 5.4). and cultured in the dark at 22°C for 3 days. After 3 days of culture, the explants were washed four or five times with sterile water and liquid induction medium (3.21 g/L B5-salt, 0.59 g/L MES, 30 g/L sucrose, 100 mg/L Timentin, 100 mg/L cefotaxime, pH 5.6) to ensure that the *Agrobacterium* was cleaned. The washed explants were transferred to induction medium and cultured under 14 h light and 10 h dark at 26°C. After 12 days of induction, soybean hairy roots emerged, which can be used for gene editing identification. Stable *Agrobacterium*-mediated soybean transformation was performed as previously described [9].

### Vector construction

The vector was constructed based on the backbone of pZHZ593 (Supplementary Data Fig. S10). The backbone contains the Basta marker for soybean transformant selection. The promoter GmM4 was a gift from Yuefeng Guan’s laboratory [32]. The DNA sequences of SpRY-STU, SpRY-STU CBE, and SpRY-STU ABE were synthesized by Genscript (Nanjing, China). All DNA fragments were constructed into the pZHZ593 backbone by the Golden Gate reaction. GmUBI3-derived SpRY-STU (pGEL678, Addgene #207511), GmM4-derived SpRY-STU (pGEL679, Addgene #207512), SpRY-STU CBE (pGEL680, Addgene #207513), and SpRY-STU ABE (pGEL681, Addgene #207514) were generated (Supplementary Data Fig. S11). For T-DNA vector construction, the oligos were synthesized by Sangon Biotech (Shanghai, China) and annealed for the Golden Gate reaction followed by DH5a *Escherichia coli* transformation. All constructs were Sanger-sequenced.

### Sanger sequencing and data analysis

Genomic DNA was extracted by the CTAB method [44]. Transgene-positive hairy roots were first identified using specific primers (Supplementary Data Table S1). Then, the DNA fragments flanking the target sites were amplified using target-site-specific primers (Supplementary Data Table S1). All the target sites were shown in the Supplementary Data Table S2. The PCR products were Sanger-sequenced and the results were analyzed using online software DSDecodeM (http://skl.scau.edu.cn/) [45]. The data were further analyzed using Microsoft Excel and GraphPad Prism 9.0 software. Differential analysis was performed using the unpaired *t*-test with two-tailed *P* value.

### Lipoxygenase activity assay

Lipoxygenase activity was measured by a colorimetric assay as described previously with minor modifications [28]. Soybean hairy roots were separately ground into powder. Then, 1 ml of lipoxygenase extract solutions 1, 2, and 3 were added to 20, 40, and 20 mg soybean hairy root powder to extract LOX1, LOX2, and LOX3, respectively. The samples were then mixed and incubated for 15 min. The clear supernatant was collected after centrifugation (12 000 rpm, 5 min, 4°C). One milliliter of substrate solution was separately added to 0.5 ml of the supernatant obtained from centrifugation as described above, mixed, and incubated for 15 min. The solution color was observed and recorded. Solution color remaining blue meant that there was no LOX1 and LOX2 activity. Solution color remaining yellow meant there was no LOX3 activity. A lighter or faded solution color indicated the presence of lipoxygenase activity.

### Detection of fatty acid components in soybean seeds

Twenty seeds of *T*_2_ homozygous *GmFAD2-1A/B* double mutant plants and control soybean variety ‘Williams 82’ were selected and ground into powder using a frozen mixing ball grinder. Two hundred milligrams of soybean seed powder was placed in a 2-ml centrifuge tube with 1 ml of sodium methanol and incubated in a 50°C water bath for 35 min. Then, 1 ml of *n*-hexane was added and centrifuged at 2000 revolutions/min for 5 min. The supernatant was placed in a special chromatographic sample bottle for gas chromatographic detection. The experiment was performed with three biological replicates. The contents of fatty acids in seeds of Williams 82 and *T*_2_ homozygous double mutant #166-11-3 are shown in Supplementary Data Fig. 8.

## Supplementary Material

Web_Material_uhae160

## Data Availability

The data underlying this article are available in the article and in its online supplementary material.

## References

[1] Tang X, Zhang Y. Beyond knockouts: fine-tuning regulation of gene expression in plants with CRISPR-Cas-based promoter editing. *New Phytol*. 2023;239:868–74. 37282668 10.1111/nph.19020

[2] Walton RT, Christie KA, Whittaker MN. et al. Unconstrained genome targeting with near-PAM less engineered CRISPR-Cas9 variants. *Science*. 2020;368:290–6. 32217751 10.1126/science.aba8853 PMC7297043

[3] Li J, Xu R, Qin R. et al. Genome editing mediated by SpCas9 variants with broad non-canonical PAM compatibility in plants. *Mol Plant*. 2021;14:352–60. 33383203 10.1016/j.molp.2020.12.017

[4] Ren Q, Sretenovic S, Liu S. et al. PAM-less plant genome editing using a CRISPR-SpRY toolbox. *Nat Plants*. 2021;7:25–33. 33398158 10.1038/s41477-020-00827-4

[5] Liu S, Zhang M, Feng F. et al. Toward a "green revolution" for soybean. *Mol Plant*. 2020;13:688–97. 32171732 10.1016/j.molp.2020.03.002

[6] Bai MY, Yuan C, Kuang H. et al. Combination of two multiplex genome-edited soybean varieties enables customization of protein functional properties. *Mol Plant*. 2022;15:1081–3. 35643862 10.1016/j.molp.2022.05.011

[7] Bao AL, Zhang CJ, Huang Y. et al. Genome editing technology and application in soybean improvement. *Oil Crop Sci*. 2020;5:31–40.

[8] Cai YP, Chen L, Sun S. et al. CRISPR/Cas9-mediated deletion of large genomic fragments in soybean. *Int J Mol Sci*. 2018;19:3835. 30513774 10.3390/ijms19123835 PMC6321276

[9] Chen X, Yang SX, Zhang YH. et al. Generation of male-sterile soybean lines with the CRISPR/Cas9 system. Crop J. 2021;9:1270–7.

[10] Do PT, Nguyen CX, Bui HT. et al. Demonstration of highly efficient dual gRNA CRISPR/Cas9 editing of the homeologous *GmFAD2-1A* and *GmFAD2-1B* genes to yield a high oleic, low linoleic and α-linolenic acid phenotype in soybean. *BMC Plant Biol*. 2019;19:311. 31307375 10.1186/s12870-019-1906-8 PMC6632005

[11] Gao Z, Chen Z, Cui Y. et al. *GmPIN*-dependent polar auxin transport is involved in soybean nodule development. *Plant Cell*. 2021;33:2981–3003. 34240197 10.1093/plcell/koab183 PMC8462816

[12] Li ZB, Cheng Q, Gan ZR. et al. Multiplex CRISPR/Cas9-mediated knockout of soybean *LNK2* advances flowering time. Crop J. 2021;9:767–76.

[13] Li M, Chen R, Jiang QY. et al. *GmNAC06*, a NAC domain transcription factor enhances salt stress tolerance in soybean. *Plant Mol Biol*. 2021;105:333–45. 33155154 10.1007/s11103-020-01091-y PMC7858558

[14] Lyu XG, Cheng QC, Qin C. et al. GmCRY1s modulate gibberellin metabolism to regulate soybean shade avoidance in response to reduced blue light. *Mol Plant*. 2020;14:298–314. 33249237 10.1016/j.molp.2020.11.016

[15] Wang L, Sun S, Wu T. et al. Natural variation and CRISPR/Cas9-mediated mutation in *GmPRR37* affect photoperiodic flowering and contribute to regional adaptation of soybean. *Plant Biotechnol J*. 2020;18:1869–81. 31981443 10.1111/pbi.13346 PMC7415786

[16] Zhang PP, Du HY, Wang J. et al. Multiplex CRISPR/Cas9-mediated metabolic engineering increases soya bean isoflavone content and resistance to soyabean mosaic virus. *Plant Biotechnol J*. 2020;18:1384–95. 31769589 10.1111/pbi.13302 PMC7206993

[17] Duan KX, Cheng YY, Ji J. et al. Large chromosomal segment deletions by CRISPR/LbCpf1-mediated multiplex gene editing in soybean. *J Integr Plant Biol*. 2021;63:1620–31. 34331750 10.1111/jipb.13158

[18] He R, Zhang P, Yan Y. et al. Expanding the range of CRISPR/Cas9-directed genome editing in soybean. *aBIOTECH*. 2022;3:89–98. 36312444 10.1007/s42994-021-00051-4 PMC9590560

[19] Feussner I, Wasternack C. The lipoxygenase pathway. *Annu Rev Plant Biol*. 2002;53:275–97. 12221977 10.1146/annurev.arplant.53.100301.135248

[20] Zhang J, Ng C, Jiang Y. et al. Genome-wide identification and analysis of LOX genes in soybean cultivar "Zhonghuang 13". *Front Genet*. 2022;13:1020554. 36276975 10.3389/fgene.2022.1020554 PMC9585170

[21] Lenis JM, Gillman JD, Lee JD. et al. Soybean seed lipoxygenase genes: molecular characterization and development of molecular marker assays. *Theor Appl Genet*. 2010;120:1139–49. 20058147 10.1007/s00122-009-1241-9

[22] Okuley J, Lightner J, Feldmann K. et al. *Arabidopsis* FAD2 gene encodes the enzyme that is essential for polyunsaturated lipid synthesis. *Plant Cell*. 1994;6:147–58. 7907506 10.1105/tpc.6.1.147 PMC160423

[23] Schlueter JA, Vasylenko-Sanders IF, Deshpande S. et al. The FAD2 gene family of soybean. *Crop Sci*. 2007;47:S-14–26.

[24] Bäurle I, Dean C. The timing of developmental transitions in plants. *Cell*. 2006;125:655–64. 16713560 10.1016/j.cell.2006.05.005

[25] Cai Y, Wang L, Chen L. et al. Mutagenesis of *GmFT2a* and *GmFT5a* mediated by CRISPR/Cas9 contributes for expanding the regional adaptability of soybean. *Plant Biotechnol J*. 2020;18:298–309. 31240772 10.1111/pbi.13199 PMC6920152

[26] Garcia MD, Nouwens A, Lonhienne TG. et al. Comprehensive understanding of acetohydroxyacid synthase inhibition by different herbicide families. *Proc Natl Acad Sci USA*. 2017;114:E1091–100. 28137884 10.1073/pnas.1616142114 PMC5321015

[27] Patterson EL, Pettinga DJ, Ravet K. et al. Glyphosate resistance and EPSPS gene duplication: convergent evolution in multiple plant species. *J Hered*. 2018;109:117–25. 29040588 10.1093/jhered/esx087

[28] Wang J, Kuang H, Zhang Z. et al. Generation of seed lipoxygenase-free soybean using CRISPR-Cas9. *Crop J*. 2020;8:432–9.

[29] Tang X, Ren Q, Yang L. et al. Single transcript unit CRISPR 2.0 systems for robust Cas9 and Cas12a mediated plant genome editing. *Plant Biotechnol J*. 2019;17:1431–45. 30582653 10.1111/pbi.13068 PMC6576101

[30] Hernandez-Garcia CM, Bouchard RA, Rushton PJ. et al. High level transgenic expression of soybean (*Glycine max*) GmERF and Gmubi gene promoters isolated by a novel promoter analysis pipeline. *BMC Plant Biol*. 2010;10:237. 21050446 10.1186/1471-2229-10-237 PMC3095320

[31] Zhang N, McHale LK, Finer JJ. Isolation and characterization of “GmScream” promoters that regulate highly expressing soybean (*Glycine max* Merr.) genes. *Plant Sci*. 2015;241:189–98. 26706070 10.1016/j.plantsci.2015.10.010

[32] Bai M, Yuan J, Kuang H. et al. Generation of a multiplex mutagenesis population via pooled CRISPR-Cas9 in soya bean. *Plant Biotechnol J*. 2020;18:721–31. 31452351 10.1111/pbi.13239 PMC7004907

[33] Bae S, Park J, Kim JS. Cas-OFFinder: a fast and versatile algorithm that searches for potential off-target sites of Cas9 RNA-guided endonucleases. *Bioinformatics*. 2014;30:1473–5. 24463181 10.1093/bioinformatics/btu048 PMC4016707

[34] Tao W, Liu Q, Huang S. et al. CABE-RY: a PAM-flexible dual-mutation base editor for reliable modeling of multi-nucleotide variants. *Mol Ther Nucleic Acids*. 2021;26:114–21. 34513298 10.1016/j.omtn.2021.07.016 PMC8413891

[35] Richter MF, Zhao KT, Eton E. et al. Phage-assisted evolution of an adenine base editor with improved Cas domain compatibility and activity. *Nat Biotechnol*. 2020;38:883–91. 32433547 10.1038/s41587-020-0453-z PMC7357821

[36] Gao C . Genome engineering for crop improvement and future agriculture. *Cell*. 2021;184:1621–35. 33581057 10.1016/j.cell.2021.01.005

[37] Zhu HC, Li C, Gao CX. Applications of CRISPR-Cas in agriculture and plant biotechnology. *Mol Cell Biol*. 2020;21:661–77. 10.1038/s41580-020-00288-9 32973356

[38] Di YH, Sun XJ, Hu Z. et al. Enhancing the CRISPR/Cas9 system based on multiple *GmU6* promoters in soybean. *Biochem Biophys Res Commun*. 2019;519:819–23. 31558318 10.1016/j.bbrc.2019.09.074

[39] Cai YP, Chen L, Zhang Y. et al. Target base editing in soybean using a modified CRISPR/Cas9 system. *Plant Biotechnol J*. 2020;18:1996–8. 32311214 10.1111/pbi.13386 PMC7540304

[40] Bai MY, Hu XC, Lin WX. et al. Development of PmCDA1-based high-efficiency cytidine base editors (ChyCBEs) incorporating a *GmRad51* DNA-binding domain in soybean. *New*. *Crops*. 2024;1:100001

[41] Huang JY, Lin QP, Fei HY. et al. Discovery of deaminase functions by structure-based protein clustering. *Cell*. 2023;186:3182–3195.e14. 37379837 10.1016/j.cell.2023.05.041

[42] Shi C, Ren Y, Liu L. et al. Ubiquitin specific protease 15 has an important role in regulating grain width and size in rice. *Plant Physiol*. 2019;180:381–91. 30796160 10.1104/pp.19.00065 PMC6501108

[43] Cheng Y, Wang X, Cao L. et al. Highly efficient *Agrobacterium rhizogenes*-mediated hairy root transformation for gene functional and gene editing analysis in soybean. *Plant Methods*. 2021;17:73. 34246291 10.1186/s13007-021-00778-7 PMC8272327

[44] Arseneau J, Steeves R, Laflamme M. Modified low-salt CTAB extraction of high-quality DNA from contaminant-rich tissues. *Mol Ecol Resour*. 2017;17:686–93. 27768249 10.1111/1755-0998.12616

[45] Xie X, Ma X, Zhu Q. et al. CRISPR-GE: a convenient software toolkit for CRISPR-based genome editing. *Mol Plant*. 2017;10:1246–9. 28624544 10.1016/j.molp.2017.06.004

