# Supplementary material for: Advancing PAM-less genome editing in soybean using CRISPR-SpRY
Source: Hortic Res. 2024 Jun 7;11(8):uhae160. doi: 10.1093/hr/uhae160 (PMC11298620; doi:10.1093/hr/uhae160)
Supplement: Web_Material_uhae160 [file web_material_uhae160.zip › 2024-05-17-Revised -Supplemental Information-Advancing PAM-less genome editing in soybean using CRISPR-SpRY.docx]

**Advancing PAM-less genome editing in soybean using CRISPR-SpRY**

Xiao Chen^1#^, Zhaohui Zhong^2#^, Xu Tang^2, 3#^, Suxing Yang^1^, Yaohua Zhang^1^, Shoudong Wang^1^, Yiqian Liu^4^, Ye Zhang^1^, Xuelian Zheng^2, 3^, Yong Zhang^2, 3*^, Xianzhong Feng^1^*^*^*

^1^ Key Laboratory of Soybean Molecular Design Breeding, Northeast Institute of Geography and Agroecology, Chinese Academy of Sciences, Changchun 130102, China;

^2^ Department of Biotechnology, School of Life Sciences and Technology, Center for Informational Biology, University of Electronic Science and Technology of China, Chengdu 610054, China;

^3^ Integrative Science Center of Germplasm Creation in Western China (Chongqing) Science City, Chongqing Key Laboratory of Plant Resource Conservation and Germplasm Innovation, School of Life Sciences, Southwest University, Chongqing, 400715.

^4^ Jilin Agricultural University, Changchun 130118, Jilin, China.

^#^ These authors contributed equally to this work.

***Corresponding authors:**

Xianzhong Feng, Email: fengxianzhong@iga.ac.cn

Yong Zhang, Email: [zhangyong916@uestc.edu.cn](mailto:zhangyong916@uestc.edu.cn)

**SUPPLEMENTAL INFORMATION**

- **Supplemental Materials and Methods.**
- **Supplemental Table 1.** Oligos used in this study.

| **Name** | **Sequence 5'-3'** | | **Description** |
| --- | --- | --- | --- |
| OL13053 | CGGATGAACACTAAGTACGATGAGAA | Forward primer for identification of transgenic soybean hairy roots | |
| OL13054 | CTCACATCGTAAACCTTGTAGTCCC | Reverse primer for identification of transgenic soybean hairy roots | |
| OL13364 | CGTAGTAATACATAACCAAAGAT | Forward primer of GmLOX1-GGA for Sanger sequencing | |
| OL13365 | TAGTTCTTTATGTAAAACGCACC | Reverse primer of GmLOX1-GGA for Sanger sequencing | |
| OL13366 | AGACACCAGCTCCACTAGTCA | Forward primer of GmLOX3-TAG for Sanger sequencing | |
| OL13367 | AAGCCTAACCTTTCCTTGTTGGT | Reverse primer of GmLOX3-TAG for Sanger sequencing | |
| OL13368 | TCTGTTTCTCAGCATGAACTCAC | Forward primer of GmDCL2a-GGT for Sanger sequencing | |
| OL13369 | GCAGCCTGTCAAGAAAACAAA | Reverse primer of GmDCL2a-GGT for Sanger sequencing | |
| OL13370 | ATGGGAGGTAGAGGTCGTGT | Forward primer of GmFAD2-1A/B-GGA for Sanger sequencing | |
| OL13371 | ATTGGCCATGCAATGAGGGA | Reverse primer of GmFAD2-1A/B-GGA for Sanger sequencing | |
| OL13372 | GCAACCAATGCAATCAAGCC | Forward primer of GmFAD2-1A/B-GGC for Sanger sequencing | |
| OL13373 | CATTACGCGGCAAATCCACT | Reverse primer of GmFAD2-1A/B-GGC for Sanger sequencing | |
| OL13374 | AAACACGCCATGAATGCAAAC | Forward primer of GmFT2a-GAT for Sanger sequencing | |
| OL13375 | GGTTTGAATAGGTTTAACTTTAAGCACAG | Reverse primer of GmFT2a-GAT for Sanger sequencing | |
| OL13376 | AATCATGGCACGGGAGAACC | Forward primer of GmFT5a-AAG for Sanger sequencing | |
| OL13377 | ACTTACCAGAGTGTAGAAGGTCC | Reverse primer of GmFT5a-AAG for Sanger sequencing | |
| OL13380 | AAGTTTGCTGATGCTTGTGGGAT | Forward primer of GmALS1-GGA for Sanger sequencing | |
| OL13381 | TCAGTACCTCGTTCTACCATCACCC | Reverse primer of GmALS1-GGA for Sanger sequencing | |
| OL13544 | AACCTATGTAAAGAATCGCAAA | Forward primer of GmLOX2-GGA for Sanger sequencing | |
| OL13545 | TGGTAACGACACAATAATCCCT | Reverse primer of GmLOX2-GGA for Sanger sequencing | |
| OL13546 | CACTGGTGTGTGGGTGATTGCT | Forward primer of GmFAD2-1A-AAT for Sanger sequencing | |
| OL13547 | AACTTTGGATTTTGGTTTTGGG | Reverse primer of GmFAD2-1A-AAT for Sanger sequencing | |
| OL13548 | AGCAAAGGAAACAATAATGGGA | Forward primer of GmFAD2-1B-AAT for Sanger sequencing | |
| OL13549 | CTTTGGATTTTGGTTTTGGGAC | Reverse primer of GmFAD2-1B-AAT for Sanger sequencing | |
| OL13550 | GTATATAAGAAAGCATAAGCCA | Forward primer of GmFT2a-TCC for Sanger sequencing | |
| OL13551 | ATTTACCCTTGGTTGGTTGACA | Reverse primer of GmFT2a-TCC for Sanger sequencing | |
| OL13552 | TAAGTGAGAAGTAATCCTAATC | Forward primer of GmFT5a-AAT for Sanger sequencing | |
| OL13553 | CTCTCTTTGGCAGTTGGCATAA | Reverse primer of GmFT5a-AAT for Sanger sequencing | |
| OL14510 | GTATCATTCAATAAGCGACCTC | Forward primer of GmCCD4-CAA for Sanger sequencing | |
| OL14511 | ATGATGGGCTTGGTTTGGTTAT | Reverse primer of GmCCD4-CAA for Sanger sequencing | |
| OL14512 | AGGACCCTTGGACTGCGTGTGG | Forward primer of GmEPSPS-GAC for Sanger sequencing | |
| OL14513 | GATGGAGGAAGAACACATAGTC | Reverse primer of GmEPSPS-GAC for Sanger sequencing | |
| OL14516 | CATCCCCAACCCCTCTAACTAT | Forward primer of GmAuxin-AAG for Sanger sequencing | |
| OL14517 | AAGGAGCTAAACATTTTGCCCA | Reverse primer of GmAuxin-AAG for Sanger sequencing | |
| OL15631 | ACCCTCATCCACCAGTCG | Forward primer of self-cleavage editing for Sanger sequencing | |
| OL15632 | GGTGCCACTTTGCTGTTT | Reverse primer of self-cleavage editing for Sanger sequencing | |
|  |  |  | |

- **Supplemental Table 2.** T-DNA constructs used in this study.

| **Target gene** | **Gene_ID** | **Purpose** | **Protospacer+PAM** |
| --- | --- | --- | --- |
| *GmLOX1/2* | Glyma.13G347600/Glyma.13G347500 | Gene knock-out | GTTGGAAAGGATACGTTCTT GGA |
| *GmLOX1/2* | Glyma.13G347600/Glyma.13G347500 | Gene knock-out | GCTCCCAAAGTTGGTAACGA AGT |
| *GmLOX3* | Glyma.15G026300 | Gene knock-out | GACACCTTTCCTTATCCTCG TAG |
| *GmLOX3* | Glyma.15G026300 | Gene knock-out | GAAAGGTGTCATTTCCTCCA AGA |
| *GmDCL2a/b* | Glyma.09G025300/Glyma.09G025400 | Gene knock-out | CCTGGATGAGCTTGGTGTTT GGT |
| *GmFAD2-1A/1B* | Glyma.10G278000/Glyma.20G111000 | Gene knock-out | ATGAAGGAACATCCGAGAAG GGC |
| *GmFT2a* | Glyma.16G150700 | Gene knock-out | ACAACGAGAGGATCCCTACT TCC |
| *GmFT2a* | Glyma.16G150700 | Gene knock-out | CTAGCCCCTGTTGTTGCTGG GAT |
| *GmFT5a* | Glyma.16G044100 | Gene knock-out | CAAAGAAACGGAGCTTGTAA AAG |
| *GmALS1* | Glyma.04G196100 | Gene knock-out | TGCCGATGATTCCCAGTAAT GGA |
| *GmEPSPS* | Glyma.03G027400 | Base editing | GGTACTGCGATGCGTCCTTT GAC |
| *GmCCD4* | Glyma.01G154900 | Base editing | CTCAACGCAGTTTCACCCCT CAA |
| *GmFAD2-1A/1B* | Glyma.10G278000/Glyma.20G111000 | Base editing | ATGAAGGAACATCCGAGAAG GGC |
| *GmFT2a* | Glyma.16G150700 | Base editing | CTAGCCCCTGTTGTTGCTGG GAT |
| *GmLOX3* | Glyma.15G026300 | Base editing | GACACCTTTCCTTATCCTCG TAG |
| *GmLOX1/2* | Glyma.13G347600/Glyma.13G347500 | Base editing | GTTGGAAAGGATACGTTCTT GGA |
|  |  |  |  |


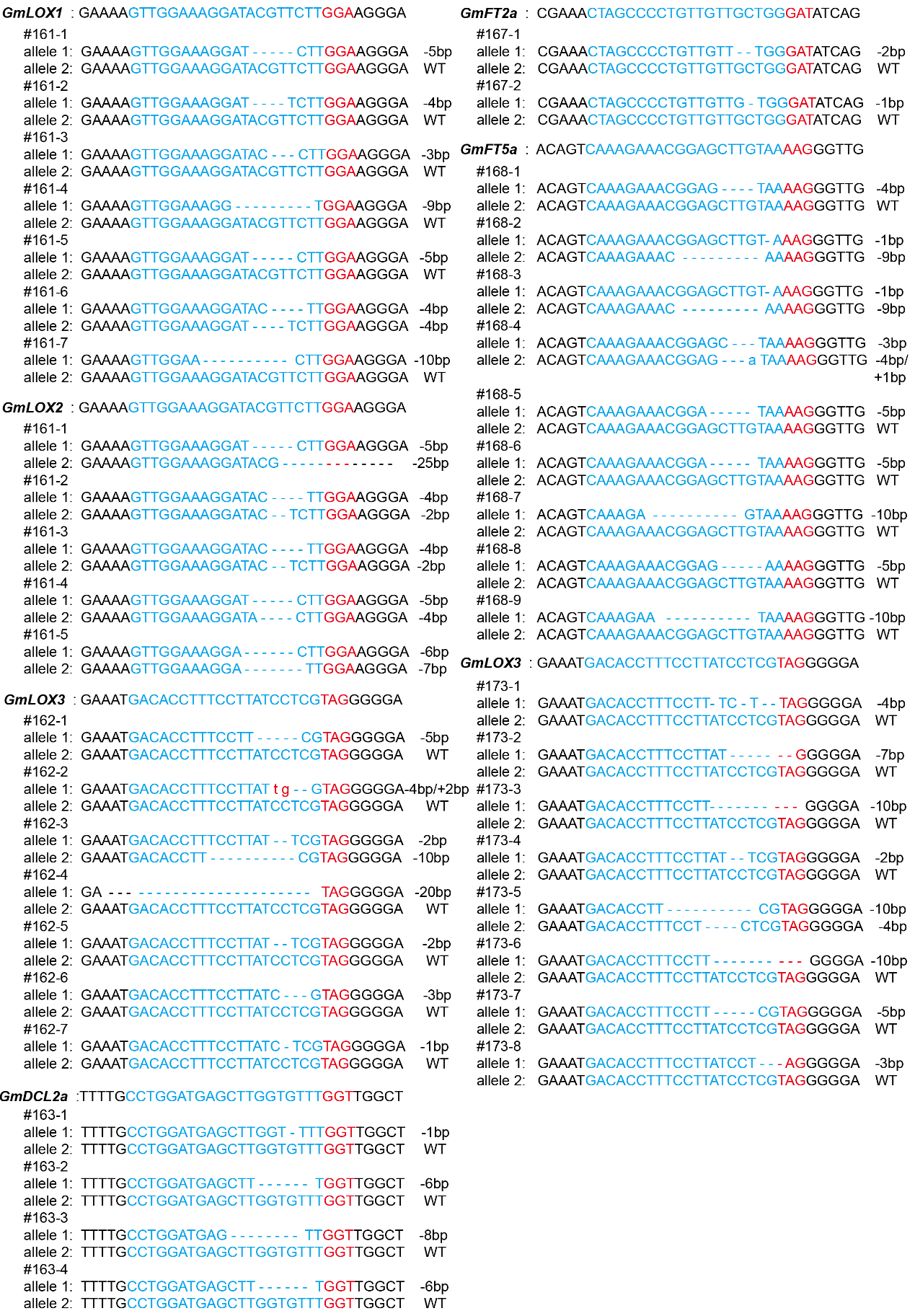


- **Supplementary Figure 1.** Genotype of SpRY mediated singular genome editing in soybean hairy roots (part 1). The protospacer was labeled in blue and the PAM was labeled in red.


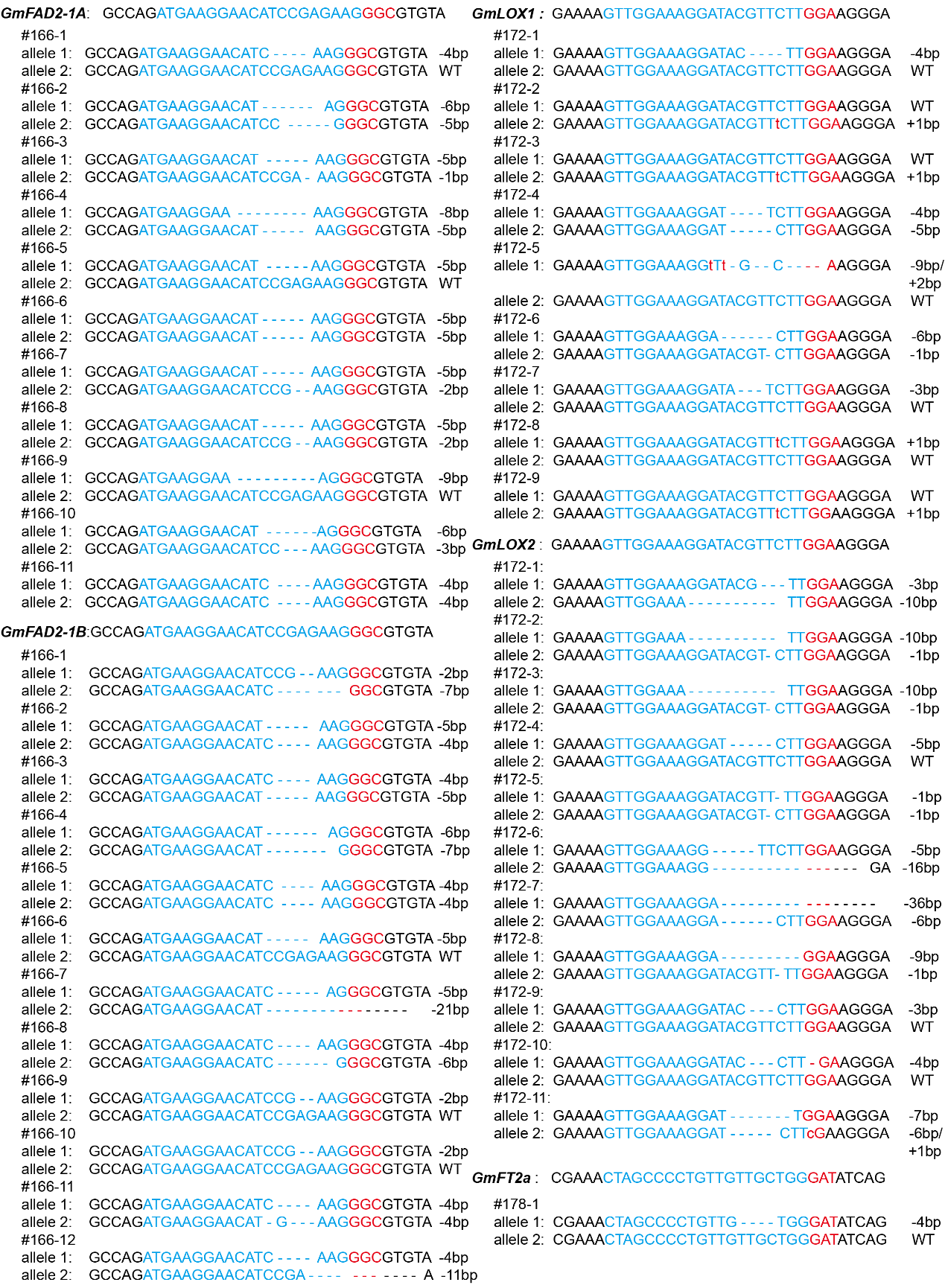


- **Supplementary Figure 2.** Genotype of SpRY mediated singular genome editing in soybean hairy roots (part 2).


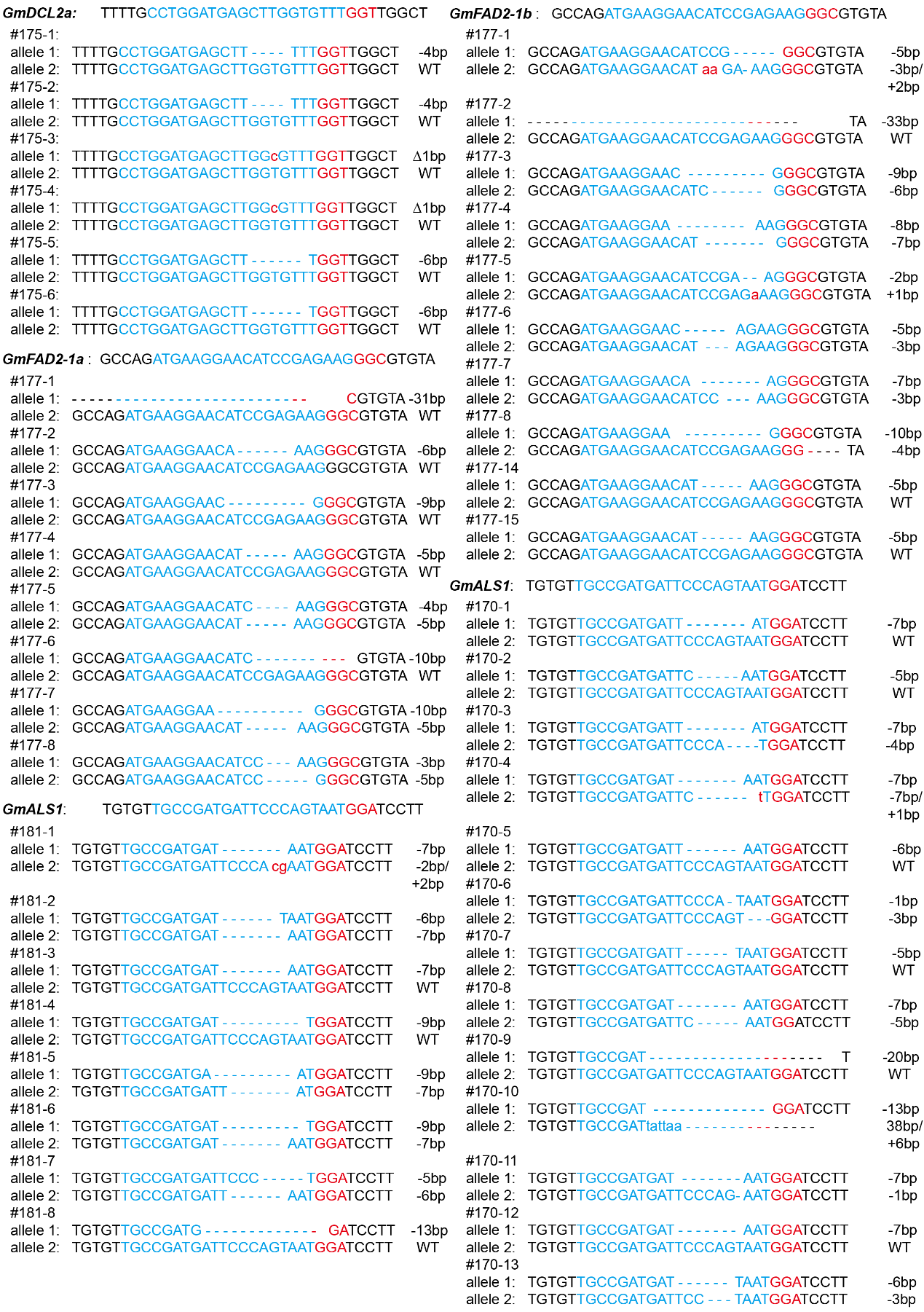


- **Supplementary Figure 3.** Genotype of SpRY mediated singular genome editing in soybean hairy roots (part 3).


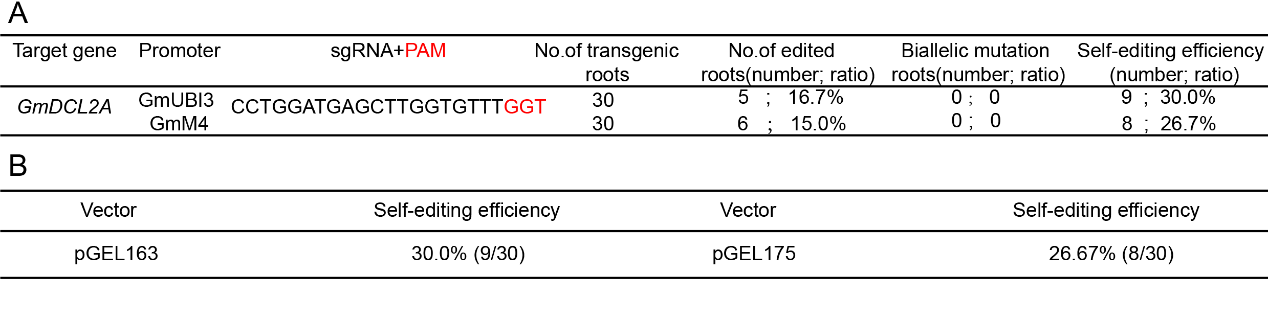


- **Supplementary Figure 4.** T-DNA self-cleavage induced by SpRY in soybean hairy roots. **(A)** Genome editing efficiency at *GmDCL2A* locus derived by two promoters. **(B)** Summary of vector self-cleavage editing for two T-DNA constructs in soybean hairy roots.


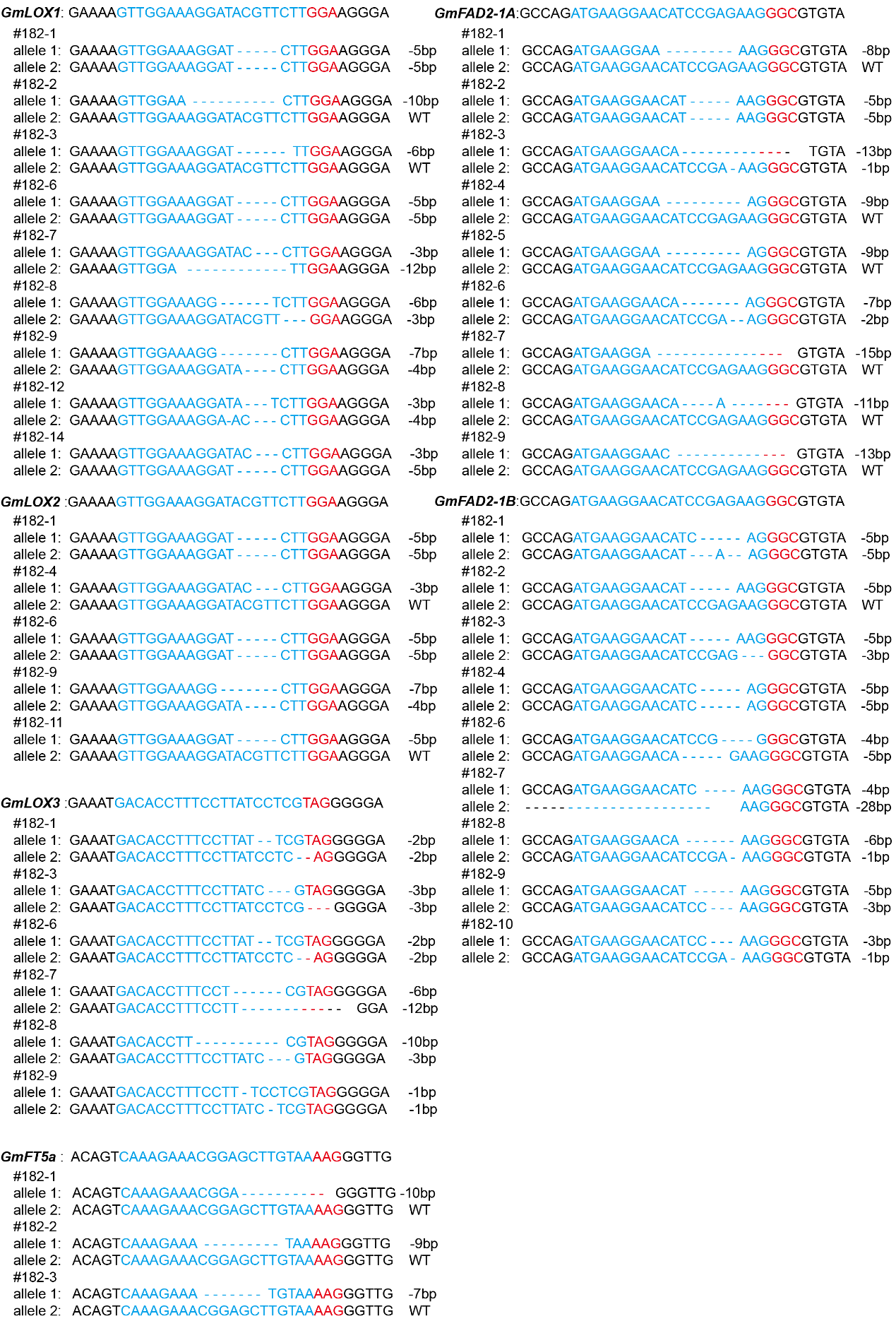


- **Supplementary Figure 5.** Genotype of SpRY mediated multiplexed genome editing in soybean hairy roots (part 1).


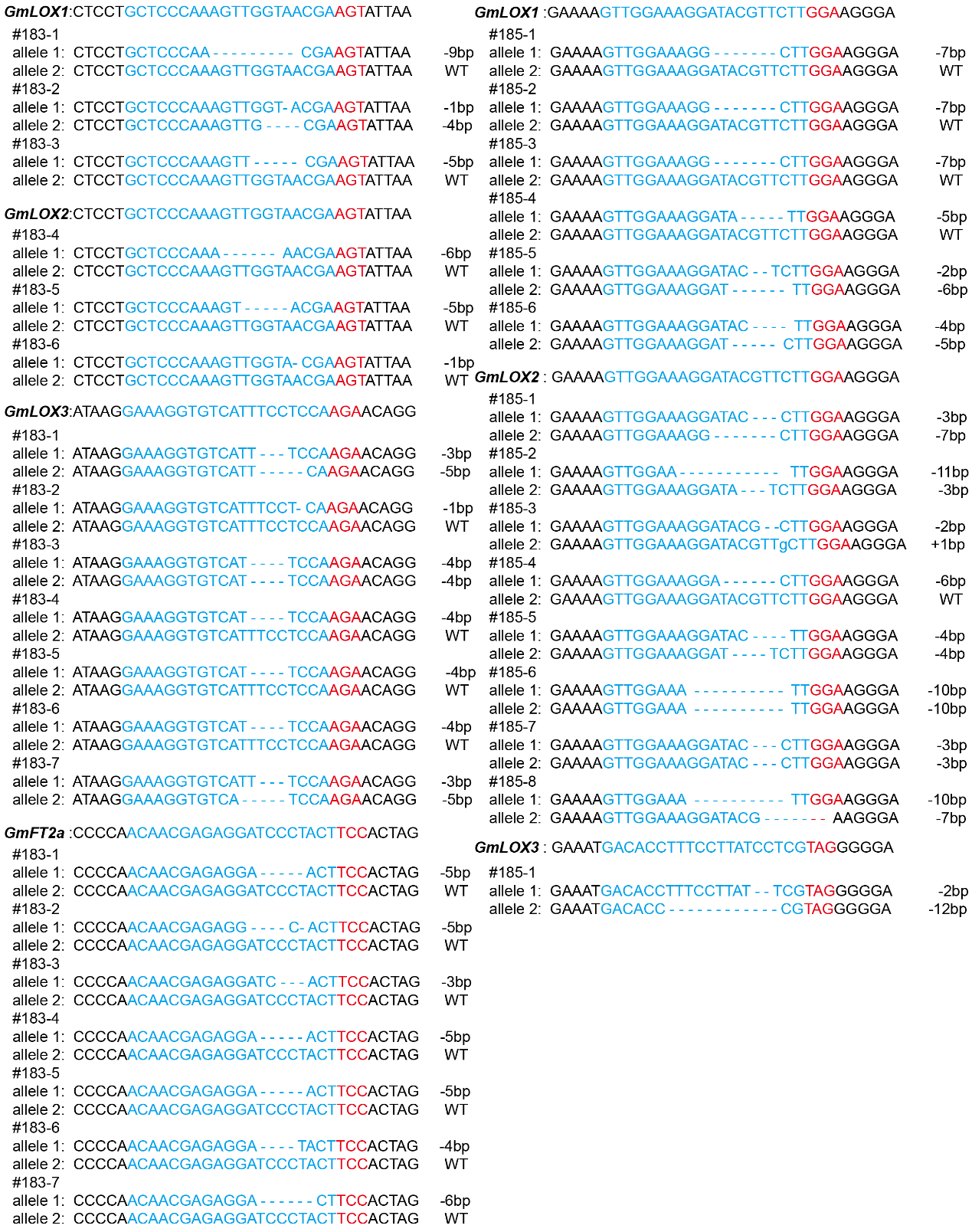


- **Supplementary Figure 6.** Genotype of SpRY mediated multiplexed genome editing in soybean hairy roots (part 2).


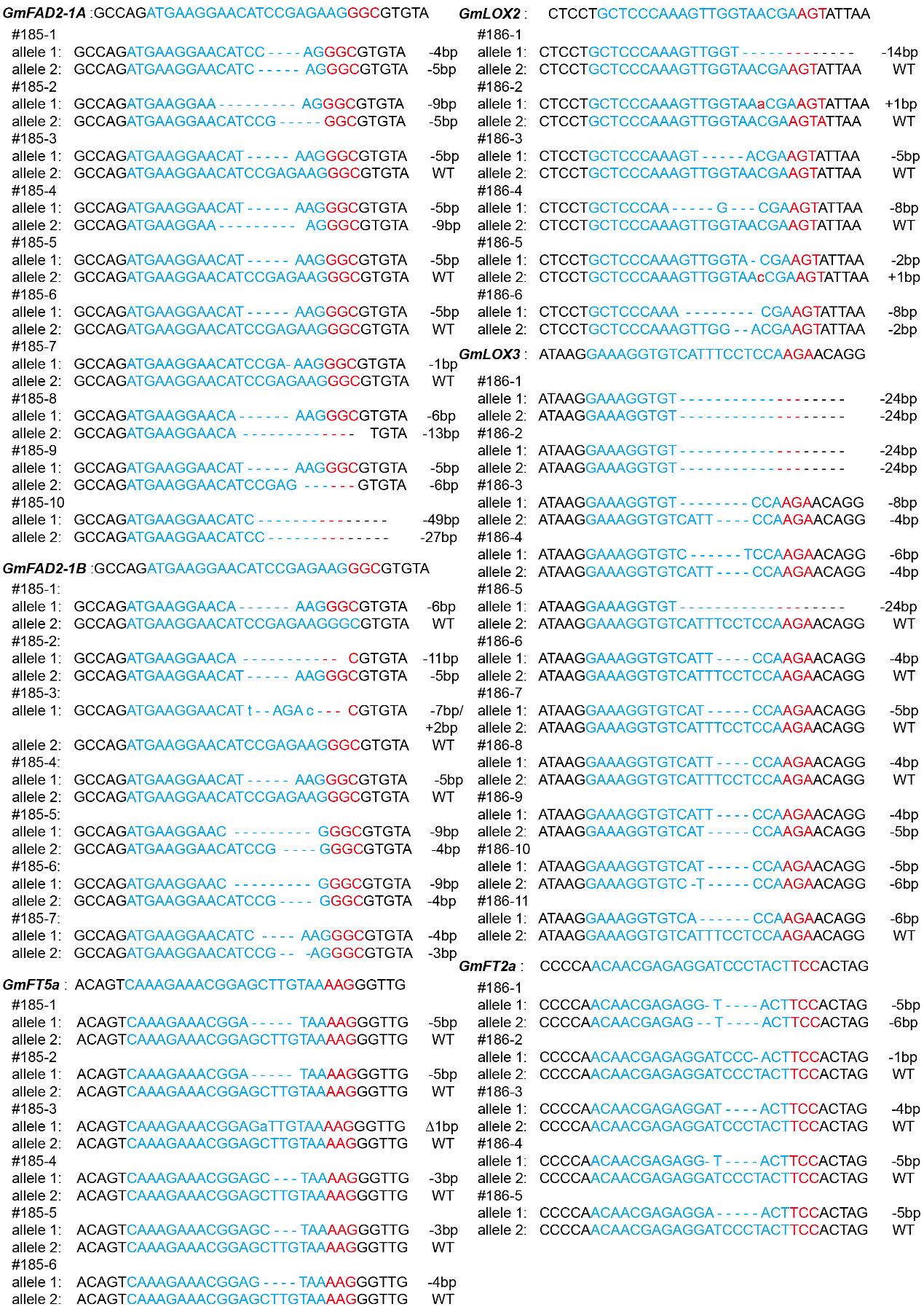


- **Supplementary Figure 7.** Genotype of SpRY mediated multiplexed genome editing in soybean hairy roots (part 3).


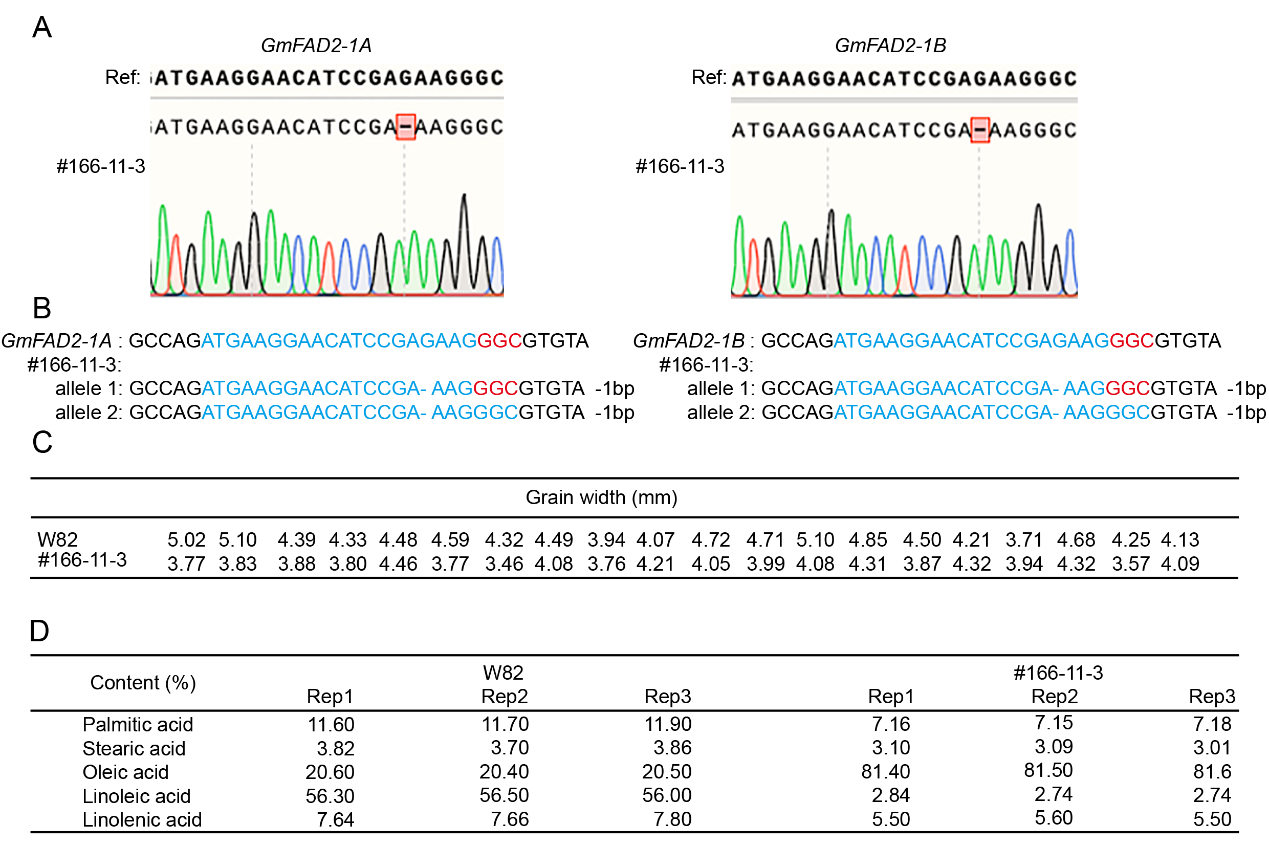


- **Supplementary Figure 8.** SpRY mediated genome editing at the *GmFAD2-1A/B* site in soybean stable line. **(A)** The sanger sequencing result of T_2_ line #166-11. **(B)** The genotype of T_2_ line #166-11-3. **(C)** The seed width of Williams 82 and T_2_ line #166-11-3 *(n=20)*. **(D)** The seeds’ fatty acid contents of Williams 82 and T_2_ line #166-11-3.


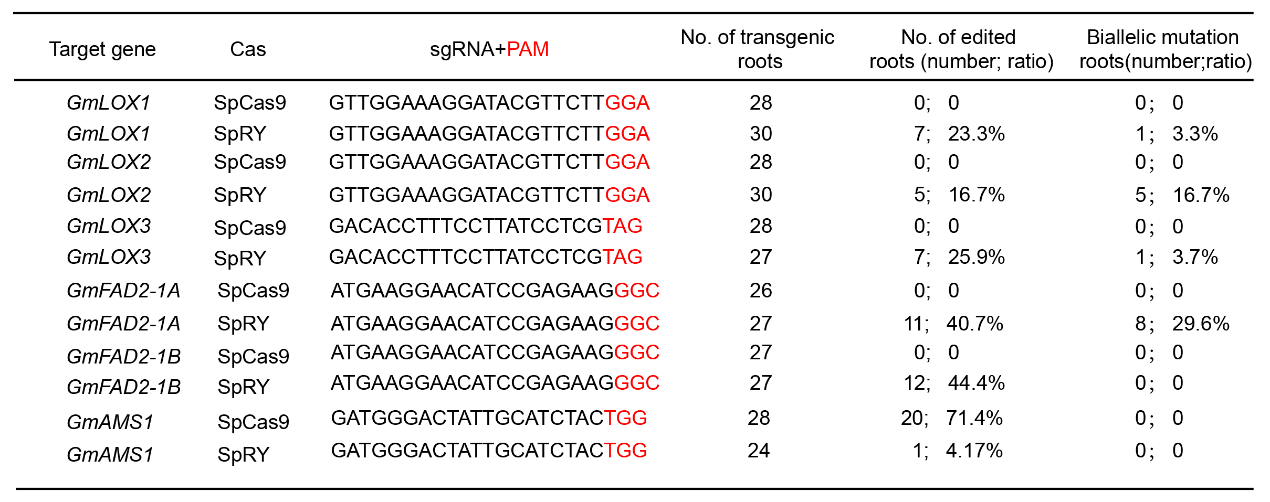


- **Supplementary Figure 9.** Comparison of SpCas9 and SpRY at editing NRN PAM sites in soybean.


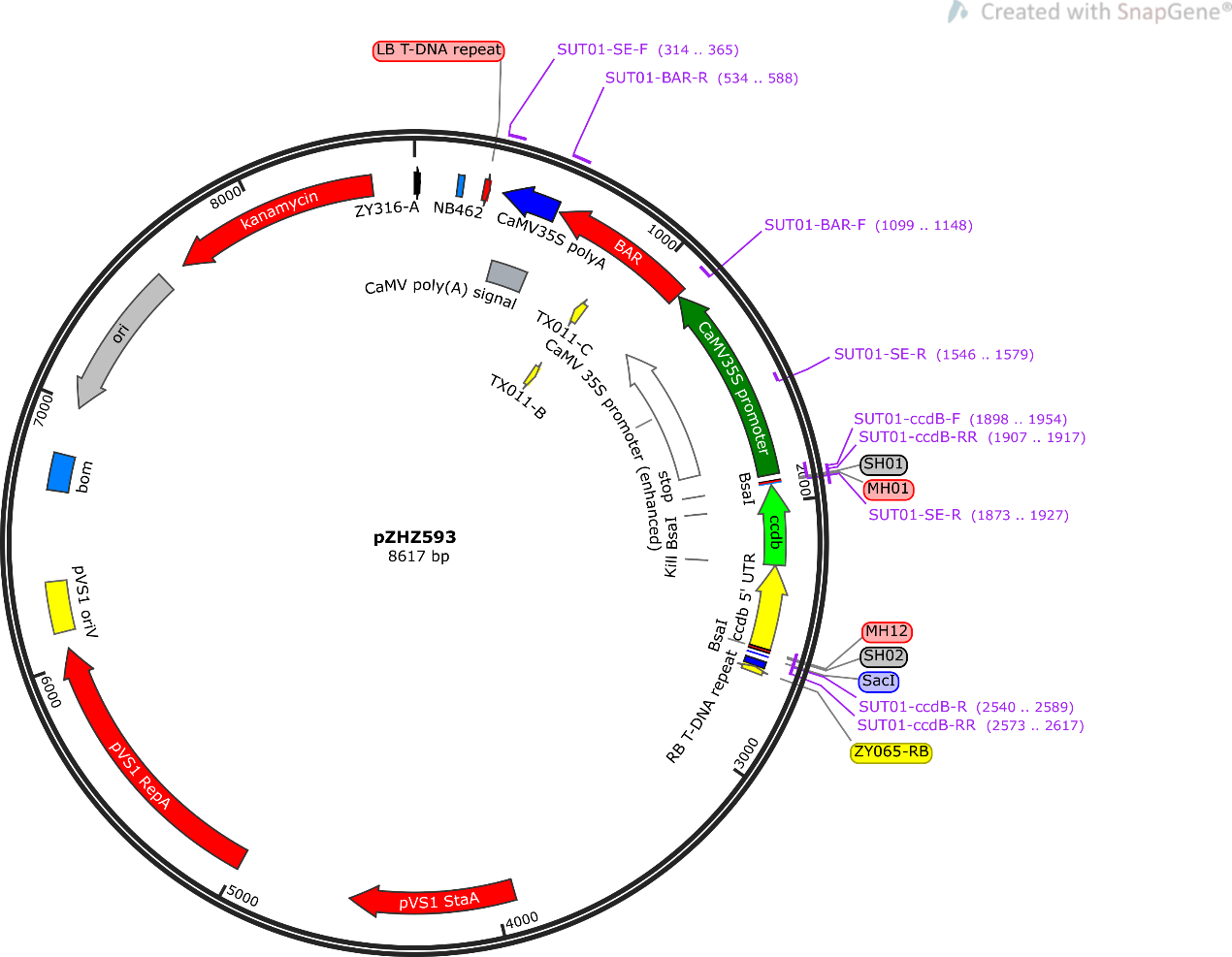


- **Supplementary Figure 10.** The schematic diagram of vector pZHZ593.


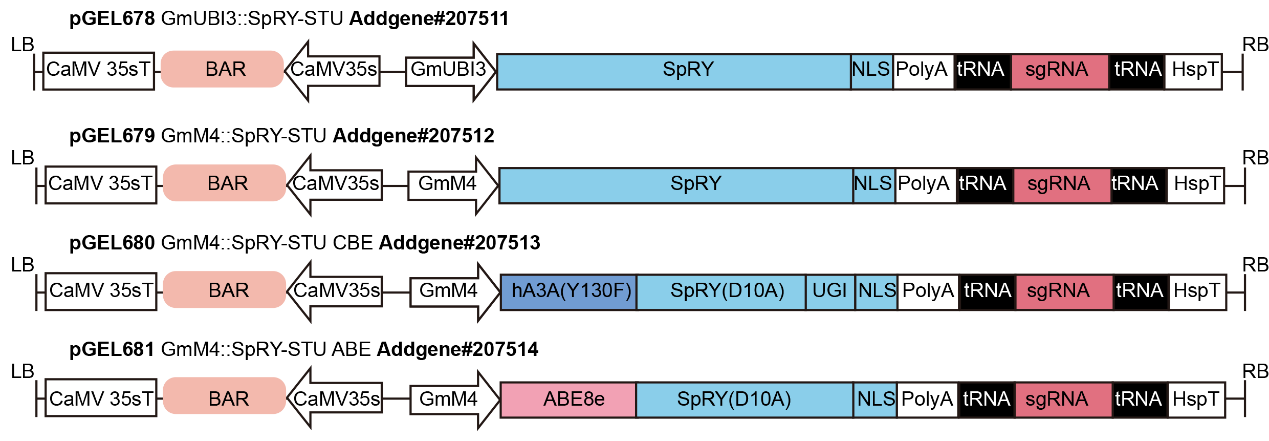


- **Supplementary Figure 11.** Schematic of the vectors of SpRY for genome editing and base editing in soybean.
